# Insulin resistance and adverse lipid profile in obese pre-pubertal South Indian children

**DOI:** 10.1186/1687-9856-2015-S1-P78

**Published:** 2015-04-28

**Authors:** Ayyavoo Ahila, Vijayakumar Vimalraj, Palany Raghupathy

**Affiliations:** 1 G. Kuppuswamy Naidu Memorial Hospital, Coimbatore, India; 2 Liggins Institute, University of Auckland, Auckland, New Zealand

## 

High prevalence of obesity, type 2 diabetes mellitus (T2DM) and the metabolic syndrome (MS) are evident among children and adults in India.[1,2] Strong evidence exists for the higher risk for insulin resistance (IR) syndrome in South Asians compared to other ethnicities, thereby demonstrating the higher risk for MS in this population.[3] MS is a risk factor for cardiovascular disease (CVD) and T2DM.[4,5] Adverse metabolic features such as dyslipidaemia are seen even in childhood and persist into adulthood with important long term implications.[6,7] Lipid profile data from pre-pubertal obese children from India is sparse and hence this has been addressed for the first time in this study, along with the relationship between lipid profile and IR in south Indian children.

Heights and weights of healthy pre-pubertal children aged 4-10 years of age were measured and body mass index (BMI) was calculated. Subjects were classified as overweight (BMI ≥85% to <95%) and obese (BMI ≥ 95%) for age and sex. Complete lipid profile as well as plasma glucose and insulin were measured in fasting venous blood samples. LDL/HDL (low & high density lipoprotein cholesterol), TC (total cholesterol)/HDL and IR by HOMA-IR (Homeostatic model assessment) were analysed.

Tests used for analysing differences between groups included: unpaired t-test for quantitative variables; comparison between groups by the non-parametric Mann-Whitney test; ANOVA for quantitative variables; Pearson coefficient of correlation for relationship between the variables and the chi square test for differences in categorical variables between groups. “P” value of <0.05 using a two-tailed test was considered significant. Data were analysed with the statistical software SPSS version 16.0.

Among the 100 participants, 98 were obese and two were overweight (M:F 51:49). Insulin resistance was observed to be higher with increased weight and BMI. A significant positive correlation was present between HOMA-IR and BMI (r = 0.291), triglycerides (r = 0.214) & TC/HDL (r = 0.229). A negative trend in correlation was found with HDL (r = -0.023).

Obese south Indian children were found to have IR and dyslipidaemia during their pre-pubertal years. Long term studies on this population and intervention measures are necessary to analyse and reduce the risk for the MS and CVD in later years.

## References

[B1] Ramachandran A Snehalatha C Vinitha R Thayyil M Sathish Kumar C Sheeba L Prevalence of overweight in urban Indian adolescent school children Diabetes research and clinical practice 2002 57 3 185 90 10.1016/S0168-8227(02)00056-6 12126768

[B2] Ramachandran A Snehalatha C Kapur A Vijay V Mohan V Das A High prevalence of diabetes and impaired glucose tolerance in India: National Urban Diabetes Survey Diabetologia 2001 44 9 1094 101 10.1007/s001250100627 11596662

[B3] McKeigue P Shah B Marmot M Relation of central obesity and insulin resistance with high diabetes prevalence and cardiovascular risk in South Asians The Lancet 1991 337 8738 382 6 10.1016/0140-6736(91)91164-P 1671422

[B4] Alberti KG Zimmet P Shaw J The metabolic syndrome--a new worldwide definition Lancet 2005 366 9491 1059 10.1016/S0140-6736(05)67402-8 16182882

[B5] Ford ES Risks for all-cause mortality, cardiovascular disease, and diabetes associated with the metabolic syndrome Diabetes Care 2005 28 7 1769 10.2337/diacare.28.7.1769 15983333

[B6] Nicklas T Von Duvillard S Berenson G Tracking of serum lipids and lipoproteins from childhood to dyslipidemia in adults: the Bogalusa Heart Study International Journal of Sports Medicine 2002 23 S1 39 43 10.1055/s-2002-28460 12012261

[B7] Srinivasan SR Frontini MG Xu J Berenson GS Utility of childhood non–high-density lipoprotein cholesterol levels in predicting adult dyslipidemia and other cardiovascular risks: The Bogalusa Heart Study Pediatrics 2006 118 1 201 6 10.1542/peds.2005-1856 16818566

